# The SMILe integrated care model in allogeneic SteM cell TransplantatIon faciLitated by eHealth: a protocol for a hybrid effectiveness-implementation randomised controlled trial

**DOI:** 10.1186/s12913-022-08293-8

**Published:** 2022-08-20

**Authors:** Sabina De Geest, Sabine Valenta, Janette Ribaut, Sabine Gerull, Juliane Mielke, Michael Simon, Jana Bartakova, Klaus Kaier, Jens Eckstein, Lynn Leppla, Alexandra Teynor, on behalf of the SMILe team

**Affiliations:** 1 Nursing Science, Department Public Health, University of Basel, Bernoullistrasse 28, 4056 Basel, CH Switzerland; 2 Academic Centre for Nursing and Midwifery, Department of Public Health and Primary Care, KU Leuven, Leuven, Belgium; 3 Department of Haematology, University Hospital Basel, Basel, Switzerland; 4 Oncology, haematology and transfusion medicine, Cantonal Hospital Aarau, Aarau, Switzerland; 5 Institute for Medical Biometry and Statistics, University of Freiburg, Freiburg, Germany; 6 Department of Internal Medicine, University Hospital Basel, Basel, Switzerland; 7 Department of Medicine I, Medical Centre – University of Freiburg, Faculty of Medicine, Freiburg, Germany; 8 Faculty of Computer Science, Augsburg University of Applied Sciences, Augsburg, Germany

**Keywords:** Care coordination, eHealth, Hybrid effectiveness-implementation study, Randomised controlled trial, Stem cell transplantation, Advanced practice nurses, Re-hospitalisations, Implementation strategies, Implementation outcomes

## Abstract

**Background:**

While effectiveness outcomes of eHealth-facilitated integrated care models (eICMs) in transplant and oncological populations are promising, implementing and sustaining them in real-world settings remain challenging. Allogeneic stem cell transplant (alloSCT) patients could benefit from an eICM to enhance health outcomes. To combat health deterioration, integrating chronic illness management, including continuous symptom and health behaviour monitoring, can shorten reaction times. We will test the 1st-year post-alloSCT effectiveness and evaluate bundled implementation strategies to support the implementation of a newly developed and adapted eICM in allogeneic stem cell transplantation facilitated by eHealth (SMILe–ICM).

SMILe-ICM has been designed by combining implementation, behavioural, and computer science methods. Adaptions were guided by FRAME and FRAME-IS. It consists of four modules: 1) monitoring & follow-up; 2) infection prevention; 3) physical activity; and 4) medication adherence, delivered via eHealth and a care coordinator (an Advanced Practice Nurse). The implementation was supported by contextually adapted implementation strategies (e.g., creating new clinical teams, informing local opinion leaders).

**Methods:**

Using a hybrid effectiveness-implementation randomised controlled trial, we will include a consecutive sample of 80 adult alloSCT patients who were transplanted and followed by University Hospital Basel (Switzerland). Inclusion criteria are basic German proficiency; elementary computer literacy; internet access; and written informed consent. Patients will be excluded if their condition prevents the use of technology, or if they are followed up only at external centres. Patient-level (1:1) stratified randomisation into a usual care group and a SMILe-ICM group will take place 10 days pre-transplantation. To gauge the SMILe–ICM’s effectiveness primary outcome (re-hospitalisation rate), secondary outcomes (healthcare utilization costs; length of inpatient re-hospitalizations, medication adherence; treatment and self-management burden; HRQoL; Graft-versus-Host Disease rate; survival; overall survival rate) and implementation outcomes (acceptability, appropriateness, feasibility, fidelity), we will use multi-method, multi-informant assessment (via questionnaires, interviews, electronic health record data, cost capture methods).

**Discussion:**

The SMILe–ICM has major innovative potential for reengineering alloSCT follow-up care, particularly regarding short- and medium-term outcomes. Our dual focus on implementation and effectiveness will both inform optimization of the SMILe-ICM and provide insights regarding implementation strategies and pathway, understudied in eHealth-facilitated ICMs in chronically ill populations.

**Trial registration:**

ClinicalTrials.gov. Identifier: NCT04789863. Registered April 01, 2021.

## Contribution to the literature

- Although eHealth-facilitated integrated care models (eICMs) for chronically ill and transplant populations show promise in RCTs, real-world translation remains challenging, calling for sustainable implementation science-powered innovation.
- Our newly-developed eHealth-facilitated ICM for allogeneic SteM cell transplantation (SMIL*e*–ICM) originated at the intersection of implementation, behavioural, and computer science methods. It will be tested using a hybrid effectiveness-implementation RCT.
- Our dual focus on implementation and effectiveness evaluation will inform optimisation of the SMILe-ICM while providing insights regarding the implementation pathway, which is understudied in chronically ill populations. Therefore, this study will inform future eICM adoption decisions.

## Background

Increasing evidence shows the potential of eHealth-facilitated integrated care models (eICMs) to improve outcomes in chronically ill populations including solid organ transplant recipients [1–6]. In chronically ill populations, empirical evidence supports the eICM’s effectiveness in view of reducing symptom severity and healthcare utilization (emergency visits and hospitalisations), as well as of increasing quality of life (QoL), survival rates and medication adherence [7–11]. Among patients with blood cancer and allogeneic stem cell transplant (alloSCT) recipients [12], several pilot studies have evaluated the acceptability, usability, and feasibility of eHealth solutions in the form of smartphone symptom management systems [13] and telehealth visits. Also, in following eICM principles to evaluate remote monitoring of adjuvant chemotherapy-related side effects on symptom burden in cancer patients, a recent European multicentre randomised controlled trial (RCT) showed significant improvements in symptom burden, distress, physical and psychological symptoms compared to patients treated according to the standard care model (*p* < 0.001) [4]. However, implementation of the eICM into routine practice remains challenging, as multilevel barriers (e.g., need for specified training, need for regulations of digital solutions by regulatory agencies) currently impede adoption [1].

Due to the chronic nature of their illness, patients with blood cancer and transplant recipients need not only biomedical, but additionally behavioural and psychosocial care. The various mechanisms through with chronically ill persons receive needed care must be addressed and coordinated across the entire care continuum [14–18]. However, as the current model of care focusses on short- to medium-term curative treatment, chronically ill persons are not well served by prevailing acute care models. Focusing primarily on biomedical aspects of care, these are generally episodic, uni-disciplinary and limited regarding self-management support and prevention [19, 20]. To improve outcomes, eHealth-facilitated integrated care models (eICMs) [1–5] correlate well with reduced symptom severity, emergency visits and hospitalisations, as well as increased quality of life (QoL), survival rates and medication adherence [7–11]. As a patient-centred system of care that addresses chronically ill persons’ complex care needs, the eICM guides care providers to improve the continuity of symptom management, relationships and communication across care settings (e.g., home, community health & hospital) and providers [21–23].

The eHealth-enhanced Chronic Care Model (eCCM) conceptually embeds the principles of chronic illness management [21, 22], encompasses eHealth and incorporates multiple CCM building blocks, i.e., self-management support, delivery systems design, clinical decision support, clinical information systems and eHealth education [23]. Further, the eCCM describes how eHealth can power each of those building blocks [24]. For example, its functions include regular monitoring and feedback regarding vital signs, symptoms and/or health behaviour of community-dwelling patients. These not only enhance self-management, but also improve communication between patients and health care providers, thereby potentially shortening reaction time in case of health deterioration [1, 2]. While eHealth is a crucial component of the eCCM, though, its human-delivered elements are equally critical. That is, while the eHealth components greatly expand patients’ capacities to recognise important changes, clinicians retain major roles in care delivery [25]. Still, as many of the patients’ concerns do not require medical expertise, the first-line clinicians managing an eICM are often nurses in advanced roles such as Advanced Practice Nurses (APNs) [26, 27].

With the increasing global burden of chronic conditions, more health care systems are recognizing the need to reengineer systems of care based on principles of chronic illness management and powering them with eHealth. This need is reflected in health care policy guidelines on ICMs and eHealth [28, 29], with eHealth also fuelled by the recent COVID pandemic [30, 31]. Further increasing the interest in assessing electronic Patient Reported Outcomes (ePROs) [32], the evidence base from trials testing the effectiveness of eICM shows promising findings [1, 7, 9, 10].

For most eHealth interventions, implementation issues are substantial: 44–67% of patients discontinue the offered eHealth tools. These problems often reflect mismatches between the technologies and their contexts, particularly their target users’ needs [33–35]. The results include low adoption rates (10–12%) [33, 35] and problems with acceptance (64–86.5%) [34]. Further, many eHealth solutions fail either to base their work on acknowledged theories or to build on empirical evidence: only 26% refer to a theory of behaviour change; and only 11.3% are evidence-based [36, 37]. For the moment, then, studies tend to report on mHealth apps’ problems with system interoperability (i.e., lack of integration with electronic health records (EHR)) [35], reimbursement structures [38], security (often resulting from insecure communication protocols), and lack of privacy policies [39]. These issues hinder adoption (e.g., a site may want to use a tool, but it may not be compatible with their EHR package) and sustainability (e.g., a site may have used a tool, but it isn’t compatible after an update) in daily clinical practice. However, even if they were accepted within a given context, their sustainability would likely be rather limited, as their developers rarely address multi-level barriers, i.e., those at the patient, health care provider and health care organizational levels [1, 2]. Therefore, implementation aspects of eHealth solutions in general and of eICMs in particular have largely been disregarded [25, 34]. Afforded little credibility, even the best are often stranded for years in the wasteland between publication and implementation.

Traversing this ‘valley of death’ will require both methodological innovation and guidance. Even where theory-based intervention development [36] and user-centred design processes are employed during development [40], no guidance is available to instruct promising interventions from trial settings past potential obstacles and pitfalls to long-term real-world use. However, one field of knowledge can help developers produce such guidance. That field is implementation science [41].

### The SMILe project: a two-phase implementation science project

Our project—the development/adaption, implementation and evaluation of an ICM in allogeneic stem cell transplantation facilitated by eHealth (SMIL*e-ICM*)—was designed to anticipate and address these methodological challenges to sustainably reengineer the follow-up care of alloSCT patients in a Swiss and German setting. AlloSCT patients are a growing group with complex care needs. In 2019, 43′581 patients across 51 countries received allogeneic stem cell transplants [42]. Despite improved long-term survival, mortality and re-hospitalisation rates remain substantial, especially in the first year post-alloSCT [43]. In particular, the first months post-alloSCT constitute the most complex phase in terms of life-threatening complications such as infections, acute graft-versus-host disease (aGvHD) and gastrointestinal complications [44–49]. Avoiding re-hospitalisations or at least shortening re-hospitalisation stays will require early detection and treatment of complications [50, 51]. To achieve that goal, alloSCT patients will need to self-manage complex therapeutic regimens; however, non-adherence is common [52–54]. While eHealth has been applied to deliver individual self-management support interventions [55, 56] or caregiver support [57] in alloSCT, the SMILe-ICM is novel. As a full-scale care model, it has the potential, when successfully implemented, to optimise care processes and ultimately improve this vulnerable group’s health outcomes.

**Fig. 1:**
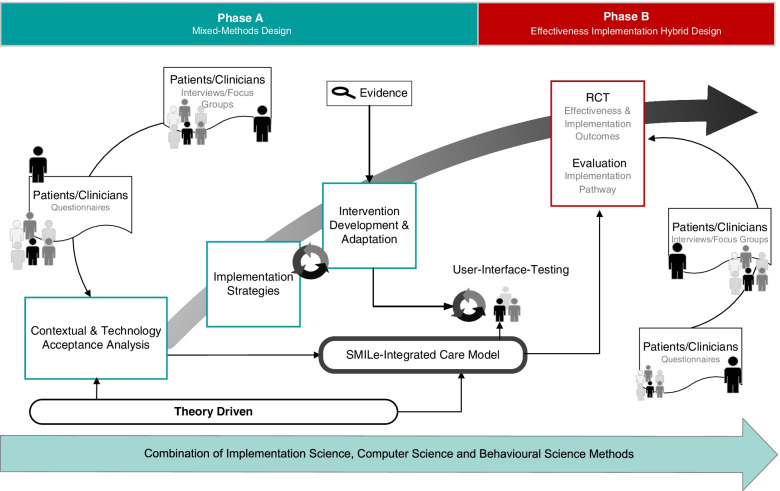
Overview of the SMILe Project’s two phases. Abbreviations. ICM = Integrated care Model; RCT = randomised controlled trial

SMILe is a two-phase implementation science project (Fig. 1). *Phase A* consisted of contextual and technology acceptance analyses. These informed the development of the SMILe-ICM and its implementation strategies for the first participating centre (University Hospital Freiburg, Germany), where implementation was successful yet no effectiveness data are available at the current time [58–62]. Phase A also guided the care model’s development and adaption for the second participating centre (University Hospital Basel (USB), Switzerland) [63], (Valenta et al. Contextspecific adaptation of an eHealth-facilitated, integrated care model and tailoring its implementation strategies – a mixed-methods study as a part of the SMILe implementation science project, Submitted). Phase A, which was completed in February 2021, [58, 60, 63], was theory-based and benefitted from continuous stakeholder involvement. *Phase B,* which will entail the implementation and testing of the SMILe-ICM in the context of the Swiss alloSCT centre (USB), is the focus of this paper.

#### The SMILe-ICM

The development and further adaption of the SMILe-ICM, which was based on a combination of behavioural, computer and implementation science methods, has been reported previously [60–62]. It is a complex, theory-based intervention, the eHealth components of which have been developed and further adapted by applying agile software development processes and a user-centred design approach [59, 62].

**Fig. 2:**
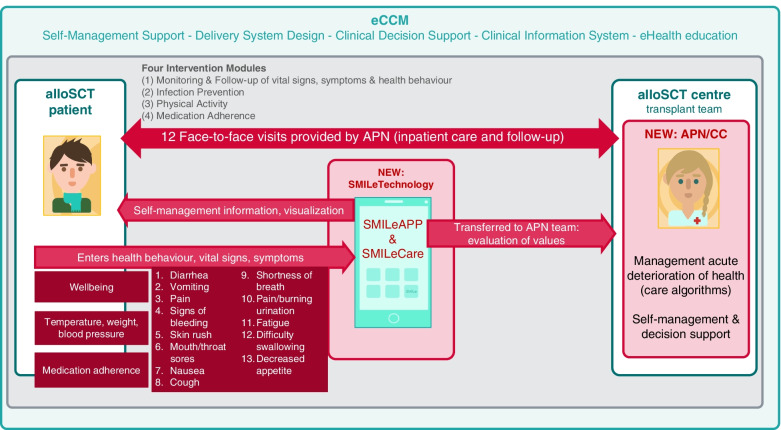
The SMILe–Integrated Care Model. Abbreviations. alloSCTs = allogeneic stem cell transplantation; APN = Advanced Practice Nurse;; CC: Care Coordinator; eCCM = eHealth enhanced Chronic Care Model

As depicted in Fig. 2 and previously reported [60–62], the SMILe-ICM is based on the five building blocks of the eCCM. It consists of *four modules* (i.e., monitoring & follow-up of vital signs, symptoms and health behaviour; infection prevention; physical activity; medication adherence). Self-management support is driven by behavioural change techniques [64]. The SMILe-ICM relies on two delivery methods: (1) a two-component technology part, i.e., a mobile app for patients (SMILeApp) and its corresponding monitoring interface for care professionals (SMILeCare); and (2) a human part, i.e., APNs acting as *SMILe care coordinators* (CCs).

1. Using the SMILeApp, patients can insert 18 relevant parameters (i.e., vital signs and PROs, see Fig. 2) on a daily basis. All data entered to the SMILeApp will be transferred to the stem cell transplant centre. With each patient’s approval, their input can be overseen by their APN/care coordinators via the SMILeCare monitoring interface. This data transfer allows the APNs to monitor, identify and act upon critical values, symptom-related issues or trends based on pre-established cut-offs and risk-adjusted care protocols. Care protocols also specify when other members of the alloSCT team (e.g., responsible physicians, nurses) will be involved [62]. Patients can also view the progress of their entered values in the SMILeApp and read up on important symptoms in the SMILeApp lexicon. In addition, each patient will receive a step counter to assess his/her daily physical activity. Added for the Swiss setting [63], medication adherence is monitored via the SMILeApp asking for daily intake of immunosuppressive medications and all other ones.
2. In addition, the APNs conduct 12 personal consultations at pre-defined timepoints starting 10 days prior to the patient’s stem cell transplantation and extending to 1 year after. The nursing visits posttransplant are planned in conjunction with the routine outpatient clinic follow-up schedule: first weekly, then monthly for stable patients. During these visits, the APN team provides intervention modules on symptom recognition and assessment, infection prevention, physical activity and medication adherence. For the full first year, considering the patients’ needs, the APNs empower and guide them to self-manage and to support behaviour change, informing and supporting them as necessary based on the course of their disease. As key members of the interdisciplinary team, the APN team promotes productive exchanges between team members and strengthens the role of nursing in the continuum of care.

#### Classification according to the medical device regulation

According to the Medical Device Regulation introduced in May 2021 [65], the SMILeApp will not be classified as a Medical Device as it can only be used to collect data, to visualise inserted values and to read lexicon information, not to provide individualised feedback regarding the entered values [66].

## Methods

### Study aims

This study has two main aims:

- **Aim 1** has two parts**:**
- **1a.** to evaluate the SMILe-ICM’s effectiveness in view of one primary outcome—re-hospitalisation rate—and seven secondary outcomes—total healthcare utilization costs, total length of inpatient re-hospitalizations, medication non-adherence, treatment burden, health-related quality of life (HRQL), quality-adjusted life year (QALY), acute and chronic GvHD incidence and grade, and overall survival rate. Regarding the primary outcome, we hypothesise that, compared to the usual-care/control group, patients receiving the SMILe-ICM will have a lower re-hospitalisation rate. As for the secondary outcome set, compared to the control group, we expect to see lower total healthcare utilization costs, shorter lengths of re-hospitalizations, lower medication non-adherence (implementation phase of adherence [67]), less treatment burden, better HRQL, higher quality-adjusted life years (QALY), and equal medical outcomes (acute and chronic GvHD incidence, overall survival).
- **1b.** to extend objective **1a** by testing for a potential wane-out of the SMILe-ICM intervention effect in view of primary and secondary outcomes during the three-month post-intervention follow-up.
- **Aim 2** is to evaluate the SMILe–ICM’s bundle of implementation strategies and assess acceptability, appropriateness, feasibility, and fidelity (implementation outcomes) as well to evaluate the implementation pathway (as viewed by patients and health care professionals).

### Study design

**Fig. 3:**
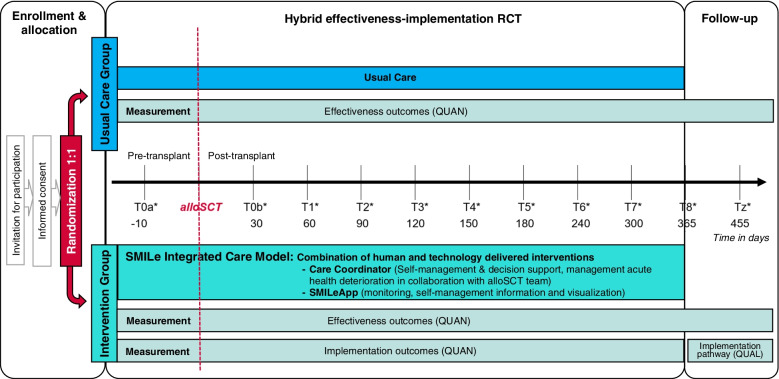
The Hybrid effectiveness-implementation RCT study design. Note. QUAL = qualitative methods; QUAN = quantitative methods; RCT = randomised controlled trial; *Timepoints data collection T0b–Tz (±7 days)

SMILe-ICM will be tested using a hybrid effectiveness-implementation randomised controlled trial (RCT) [68]. While the evaluation of the novel SMILe-ICM’s effectiveness regarding primary and secondary outcomes will be our central concern, the chosen design will also allow us to evaluate the bundle of implementation strategies by assessing implementation outcomes and the implementation pathway. This approach will allow us not only to plan further SMILe-ICM scale-ups to other settings, but also to inform the fine-tuning of the intervention and our bundle of implementation strategies. Figure 3 highlights the SMILe hybrid design, including its enrolment, allocation, randomization (see also below) and measurement time-points. The SMILe study was registered via ClinicalTrials.gov: NCT04789863 and approved by the responsible ethics committee (Ethics Committee Northwest and Central Switzerland (EKNZ: 2021–00202)). We describe the methods of the evaluation of the SMILe-ICM based on the SPIRIT guidelines [69].

### Context and targeted sites

In Switzerland, nearly 250 alloSCTs are performed annually. Roughly 110 of these take place at the USB, which is the largest alloSCT program in Switzerland [70]. The USB has a designated alloSCT unit and outpatient clinic, as well as a specialised alloSCT medical and nursing staff. Patients are hospitalised about 10 days before their transplantation. After it, depending on their state of health, they remain in hospital an average of 25 days. As noted, post-transplant monitoring and care follow an outpatient follow-up schedule. Depending on the patient’s state of health, post-transplant care then can be transferred to closely-collaborating external centres. Patients from the Switzerland’s Italian-speaking region (Ticino) receive their follow-up care solely at the haematology departments closest to their homes.

One issue revealed by the current study’s contextual analysis (Phase A) is that, once patients are discharged, they receive a low mean level of chronic illness management including limited self-management support. The clinician group most qualified to remedy this shortfall would be the APNs. However, while they are involved in immediate post-transplant care and discharge planning, longer-term post-transplant management is not yet organised as a multidisciplinary topic.

An academic service partnership between the USB and the University of Basel’s Institute of Nursing Science (INS) provides the infrastructure that supports the SMILe project. Two of the SMILe investigators (SV, JR) work in joint INS/USB appointments.

According to the Swiss Federal Law on Health Insurance, health insurance covers all allowable costs of medical treatment and hospitalisation. The patient’s deductible amounts and co-payments can only be used towards these costs [71]. This system creates a problem for eHealth innovations. While eHealth is promoted at the policy level in Switzerland, major heterogeneity in operational systems hinders easy roll-out of eHealth interventions [72, 73]. And, although digitization financing is promoted within the “Swiss eHealth Strategy 2.0 2018-2022” [29], no nationwide compensation models currently enable billing and remuneration for telemedicine [73]. This means that no provision currently exists to compensate either eHealth nor APNs for their work monitoring alloSCT recipients’ daily reports. Health-Apps can currently neither be prescribed nor paid for by health insurances [74], as it is already the case in other countries [75].

### Study participants, recruitment, and randomization

#### Sample

To reach Aims 1 and 2, both patients and members of the alloSCT team will be recruited respectively as this study’s subjects and as its data collectors. For aim 1, all adult alloSCT patients scheduled for transplantation at the USB Department of Haematology will be invited to participate in this study. They will be eligible if (1) they have basic German language proficiency and basic computer literacy; (2) they are to be both transplanted and followed-up at USB; (3) they have internet access at home; (4) they are able to carry out self-management tasks. Patients will be excluded if (1) they have cognitive dysfunction, hearing impairment or any handicap precluding use of the necessary technology and/or active participation in face-to-face visits; (2) they are scheduled to receive their second alloSCT within 1 year.

A purposive sample of alloSCT team members (i.e., haematologists, nurses, psycho-oncologists, managers) involved in in-patient and follow-up care will be invited to participate in surveys and focus group interviews. These will be used to evaluate the bundle of implementation strategies by respectively the implementation outcomes and the evaluation of the implementation pathway (Aim 2).

#### Sample size determination for aim 1

In order to determine an appropriate patient sample size, we considered the USB’s baseline hospitalisation event rate per patient year. As exact patient year data are not available, we assumed that in any given year, each transplant patient year receives roughly 0.5 patient years of care. We based our calculations on Schenkel et al.’s [6] study, which used patient years (number of hospitalisation events per patient year (HEPPY)) as an outcome for an intervention in lung transplantation.

The hospitalisation rate consists of two elements: the number of hospitalisations and the period (in years) over which patients are followed up. We conducted simulations using the paramtest software package [76], computing 10′000 iterations of the chosen parameters. Based on the USB’s available data, we set the hospitalization events per patient year to 1.56. The study’s observation period is 15 months (1.25 years). We determined the minimum sample size using the inverse of the effect size in Schenkel et al. [6], i.e., 1.79 [1.31–2.43]. Following Olivier et al.’s guidance [77], we aimed for the upper bound of a small effect size. The simulation is based on a binomial distribution with a shape parameter of 4. Assuming an α level of 0.05, a power of 80% and an effect size of 1.79, a minimum sample size of 52 patients (26 per group) was determined. Based on a refusal rate of 10% and a 15% drop out rate, then, we will aim to recruit a total of 80 patients (40 per group). Considering that approximately 70 adults per year receive *both alloSCT and follow-up* at USB, recruitment will take a minimum of 16 months.

#### Recruitment

The project leader of the SMILe project at USB (SV, shared first author), will screen potentially eligible participants. Eligibility criteria (see above) were kept as broad as possible to more closely approximate “real” patients. Following review of prospective participants’ medical records, those who meet the study’s inclusion criteria will be invited to participate. Written informed consent forms will be obtained from all participants before enrolment. These will be stored safely in the Investigator Site File. Patients have the right to withdraw from the study at any time without consequences.

#### Randomization process

**Fig. 4:**
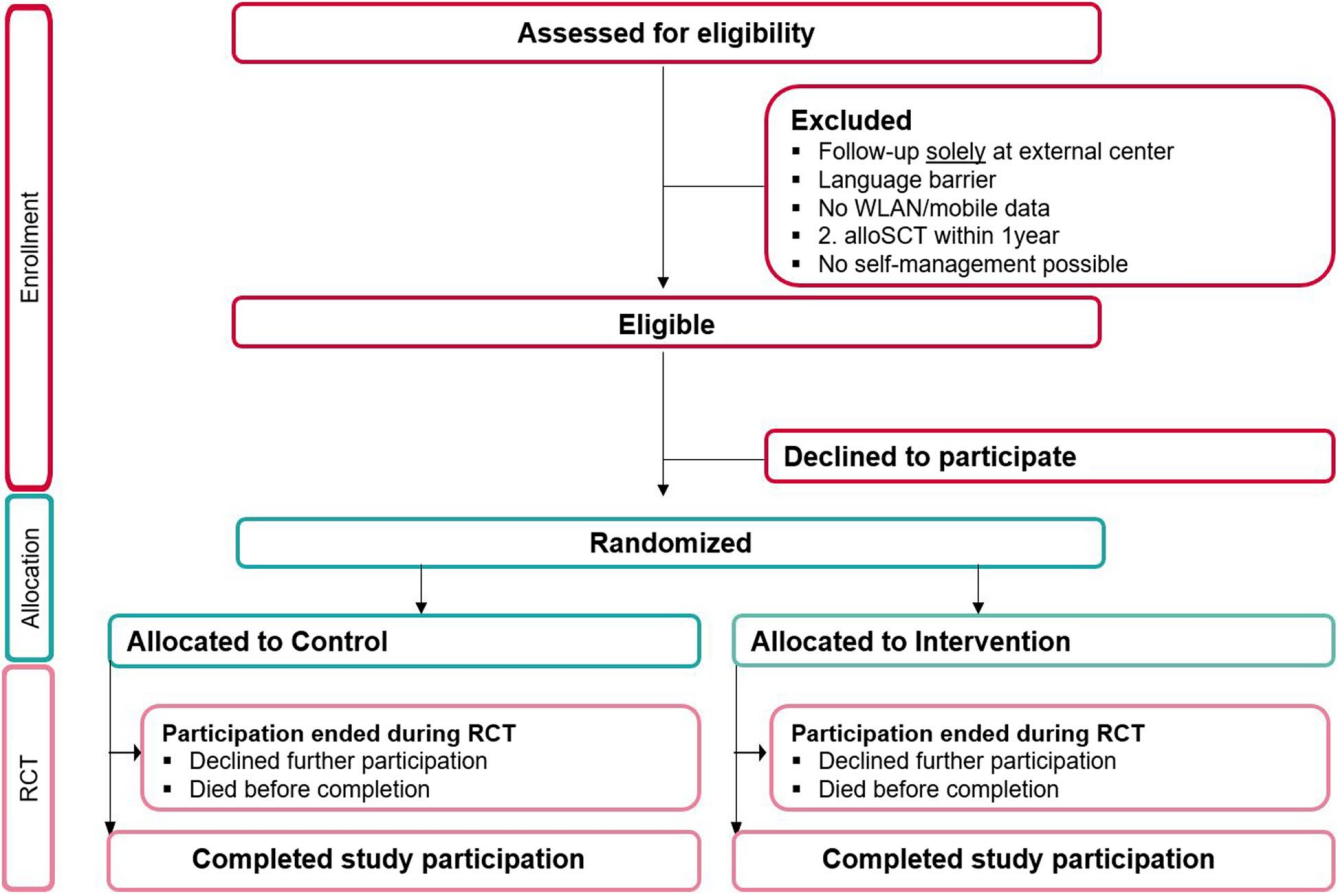
Consort flowchart

Intervention patients will receive standard care plus the SMILe-ICM. The usual care group (UCG) will receive standard care. Patients will be randomly allocated 1:1 to the intervention group (IG) or UCG at hospitalisation, i.e., approximately 10 days pre-transplant. Randomization will be stratified by risk group according to age (>/< 65 years), gender (male/female) and living alone (yes/no). The concealed randomization procedure will be implemented via the secuTrial® web-based clinical data management system provided by the USB’s Clinical Trial Unit. After randomization and allocation, no further blinding will be feasible in any group. The study’s duration will be from approximately 10 days pre-transplant to one-year post-transplant, with 3 months’ follow-up after cessation of the intervention period to test for a wane-out effect. A CONSORT flowchart for patients is presented in Fig. 4.

#### Sampling for aim 2

To evaluate the implementation outcomes and pathway (Aim 2), 10–15 health care professionals on the alloSCT care team (i.e., senior and assistant physicians, APNs, professional nurses, nurse leaders and management, psychooncologist, nutritionist) will be invited to participate in the focus group interviews. In addition, 10 individual patient interviews will be conducted between June and December 2022 with all participants’ written consent.

### Usual care and intervention group

#### Usual care group (UCG)

Usual care (see context description above) will be given to patients allocated to the UCG*.* If these participants report symptoms or any concerns, the research assistant will encourage them to contact their physicians.

#### Intervention group (IG)

The intervention, i.e., the SMILe-ICM as described above and shown in Fig. 2, will be delivered by eHealth and care coordinators (CCs). The four CCs will be specially-trained APNs. In addition to holding Master’s degrees in nursing, all CCs will be experienced alloSCT experts. Prior to study start, a six-day training segment has been provided by the USB’s SMILe project leader (SV, shared first author) in March 2021. The IG participants will receive care as usual *PLUS* the SMILe-ICM (as described above).

#### Adaptations of the SMILe-ICM intervention to the Swiss setting

The SMILe-ICM was initially developed for the German setting [62], then further developed and adapted to the Swiss setting using information gathered by the contextual analysis [63]. The Swiss adaptations to the SMILe-ICM consisted mainly of technology-based changes to guarantee the SMILe technology’s interoperability with the USB’s information systems and care processes regarding alloSCT patients. A number of adaptions within the Swiss setting’s clinical processes and structures were also necessary. Based on our theoretical framework (the Framework for Reporting Adaptations and Modifications–Expanded (FRAME) [78]), further adaptation required context-specific tailoring of the intervention’s delivery timepoint, and modification both of content (e.g., adding iOS versions of the SMILeApp and EM device, providing less self-management support in the inpatient setting) and of care algorithms based on Swiss clinical requirements.

#### SMILe implementation strategies

**Fig. 5:**
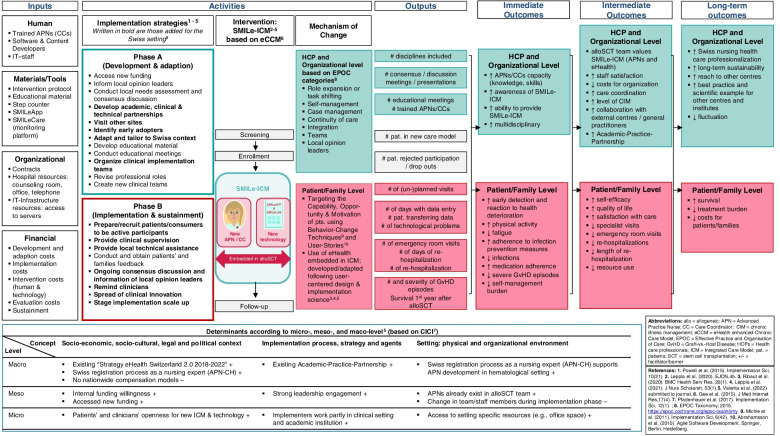
The SMILe LOGIC model

Implementation strategies to facilitate the uptake and successful implementation of the SMILe-ICM were contextually tailored to the USB setting by merging our previous experience from University Hospital Freiburg (Germany) [59] with results from the Swiss version’s contextual analysis and adaption phase, which included multiple stakeholders’ perspectives. Following the Expert Recommendations for Implementing Change (ERIC) guidelines [79, 80], we named implementation strategies and reported adaptions using the Framework for Reporting Adaptations and Modifications to Evidence-based Implementation Strategies (FRAME-IS) [81]. In Phase A of the SMILe project, a number of implementation strategies had already been applied and formulated based on the synthesis of the key contextual findings for the first participating centre [59]. As shown in Table 1, further implementation strategies—this time related to the adaption the SMILe-ICM and its evaluation phase—were further elaborated to increase the proposed implementation efforts’ acceptability, appropriateness, and feasibility. Tailoring of implementation strategies were based on multi-stakeholder input and integration of contextual analysis information. As shown in Fig. 5, a bundle of context-specific implementation strategies formulated for the first participating centre (e.g., access new funding, conduct educational meetings) [59], have been further adapted and extended to match the Swiss setting’s adaptation phase A (e.g., develop academic, clinical & technical partnerships, visit other sites, adapt and tailor to Swiss context). For Phase B, specific implementation strategies have been added (e.g., providing clinical supervision, initiating and participating in moderating ongoing consensus discussions, reminding clinicians) and generally guiding the implementation and sustainment phase within the clinical setting.

**Table 1:** The SMILe contextually adapted implementation strategy bundlerelated toSMILe project’s phases A and B

Pre-Phase	Phase A	Phase B	Sustainment
**Access new funding**	Conduct local needs assessment and consensus discussion	**Prepare/recruit patients/consumers to be active participants **	
**Develop aca- demic/clinical partnerships**	Develop academic, clinical & technical partnerships	**Ongoing consensus discussion and information of local opinion leaders**	
**Inform local opinion leaders**	**Visit other sites**	**Provide clinical supervision**	
	**Identify early adopters**	**Provide local technical assistance**	
	Organize clinical implementation teams	**Conduct and obtain patients’ and families feedback**	
	**Adapt and tailor to Swiss context**	**Remind clinicians**	
	Develop/adapt educational material	**Spread of clinical innovation**	
	Conduct educational meetings	**Stage implementation scale up**	
	Revise professional roles		
	Create new clinical teams		

***Note.*** Contextually adapted implementation strategies according to ERIC guidelines [79, 80] with respect to SMILe project’s phases A (development & adaption) and B (implementation & evaluation). In bold are those added additionally for the Swiss setting (second participating centre, Phase A and B) from the German setting (first participating centre, Phase A) [59]

#### The SMILe ICM’s logic model

In accordance with Smith and colleagues’ “Implementation Research Logic Model” [82], Fig. 5 summarizes the SMILe-ICM in a logic model. While providing information in view of *inputs, activities, outputs, outcomes* and *impact,* this logic model also provides assumptions underlying this complex intervention and highlighting the hypothesised pathways via which we hope the SMILe-ICM and implementation strategies will achieve their intended outputs and outcomes.

#### Data collection and management

Data will be collected at pre-defined time points from April 2021 until March 2024 (T0-Tz, see Table 2) by trained APNs using a standardised method in the IG and by one research assistant (using the same method) in the UCG. SMILe data will be de-identified with access limited only to authorized research study team members.

**Table 2:** Variables, measurement and data collection points

		***Measurement timepoints (T0 – Tz) and days post-Tx***										
**Variables**	**Measurements**	**T0a**	**T0b**	**T1**	**T2**	**T3**	**T4**	**T5**	**T6**	**T7**	**T8**	***Tz***
		**−10**	**30**	**60**	**90**	**120**	**150**	**180**	**240**	**300**	**365**	***455***
**Primary outcome**												
Re-hospitalisation rate (number of re-hospitalisations per patient year)	FIMA**©** (10 Items) [83] Medical Records, CRF											
**Secondary outcomes**												
Healthcare utilisation costs	FIMA**©** (10 Items) [83]			**X**	**X**	**X**	**X**	**X**	**X**	**X**	**X**	**X**
	Swiss standardised unit costs [84, 85]			**X**	**X**	**X**	**X**	**X**	**X**	**X**	**X**	**X**
Length re-hospitalizations	FIMA**©** (10 Items) [83] Medical Records, CRF			**X**	**X**	**X**	**X**	**X**	**X**	**X**	**X**	**X**
Medication adherence (implementation & persistence dimension [67])	Electronic Monitoring: MEMS® Button [86]											
	BAASIS**©** (implementation 4 items; persistence: 1 item) [87]		**X**	**X**	**X**	**X**	**X**	**X**	**X**	**X**	**X**	**X**
Treatment burden	PETS**©** (60 items) [88]	**X**			**X**			**X**			**X**	
Health-related Quality of Life	EQ-5D-5L**©** (5 items) [89]	**X**	**X**	**X**	**X**	**X**	**X**	**X**	**X**	**X**	**X**	**X**
Quality-Adjusted Life Year (QALY)	EQ-5D-5L**©** (5 items) [89], value set for Germany EQ-VT v. 2.0 (116)	**X**	**X**	**X**	**X**	**X**	**X**	**X**	**X**	**X**	**X**	**X**
Incidence/grade chronic and acute GvHD	Medical record, CRF		**X**	**X**	**X**	**X**	**X**	**X**	**X**	**X**	**X**	
Overall survival rate	Medical record, CRF		**X**	**X**	**X**	**X**	**X**	**X**	**X**	**X**	**X**	**X**
Conditioning regimen	Medical record, CRF	**X**										
Donor match/type	Medical record, CRF	**X**										
Disease	Medical record, CRF	**X**										
Disease status at transplant	Medical record, CRF	**X**										
If death: Date and cause of death	Medical record, CRF		**X**	**X**	**X**	**X**	**X**	**X**	**X**	**X**	**X**	**X**
If relapse: Date of relapse	Medical record, CRF		**X**	**X**	**X**	**X**	**X**	**X**	**X**	**X**	**X**	**X**
**Demographics**												
Age	Medical Record, CRF	**X**										
Sex	Medical Record, CRF	**X**										
Education	Questionnaire (4 items)	**X**										
Living alone	Questionnaire (1 item)	**X**										
**IMPLEMENTATION OUTCOME DATA (from intervention group / care coordinator team)**												
Acceptability	AIM (4 items) [90]	**X**	**X**	**X**	**X**	**X**	**X**	**X**	**X**	**X**	**X**	
Appropriateness	IAM (4 Items) [90]	**X**	**X**	**X**	**X**	**X**	**X**	**X**	**X**	**X**	**X**	
Feasibility	FIM (4 items) [90]	**X**	**X**	**X**	**X**	**X**	**X**	**X**	**X**	**X**	**X**	
Technology feasibility	N (days with data entry), N (technical problems)											
Intervention fidelity	N (visits), Minutes (visits), N (Behavioural Change Techniques) compared to protocol											
Intervention fidelity	5% of intervention sessions will be randomly selected to be audiotaped and checked for protocol congruence											

*Abbreviations. AIM* Acceptability of Intervention Measure, *BAASIS* Basel Assessment of Adherence to Immunosuppressive Medication Scale, *CRF* case report form, *FIM* Feasibility of Intervention Measure. *IAM* Intervention Appropriateness Measure, *MEMS* Medication Event Monitoring System, *PETS* Patient Experiences with Therapy and Self-management, *Tx* transplantation

#### Variables and measurement

Effectiveness outcomes (Table 3) will be assessed in the IG and UCG at the time of inpatient stay and during regularly scheduled outpatient appointments. After completion of the intervention period, a three-month follow-up is planned to assess sustained health outcomes by further assessing effectiveness outcomes in both groups (see also below).

**Table 3:** Effectiveness outcomes

Outcome variable(s)	Measure(s) and data collection procedures	Data source and reporter (when applicable)
**Primary Outcome**		
Re-hospitalisation rate	Number of events after the initial post-alloSCT discharge per patient in the first year post-alloSCT	EHR
**Secondary Outcomes**		
Healthcare utilization costs	(1) Calculated from the payers’ perspective based on standardised unit costs of resources in Switzerland [84, 85, 91]	EHR
	(2) Medical records and an adapted version of the generic FIMA© self-reporting questionnaire for elderly persons [83]: 7 items, asking retrospectively for number of visits to physicians, days/hours of ambulatory healthcare visits, days/hours of home care services received, days/hours of support by family caregivers, type(s) and duration(s) of rehabilitation therapy, reason(s) and duration(s) of inpatient days (including intensive care unit stays and/or emergency room visits), and current type of insurance.	Paper survey (pat.)
Length re-hospitalizations	(1) The total length of inpatient re-hospitalizations in the first year after alloSCT is the total number of hospitalized days (planned and unplanned) after initial post-alloSCT discharge until end of study, like reported in all medical reports and the generic FIMA© self-reporting questionnaire for elderly persons [83]	Paper survey (pat.) and EHR
Medication adherence (implementation & persistence dimension [67])	(2) Implementation & persistence dimension [67] will be assessed using the Basel Assessment of Adherence to Immunosuppressive Medication Scale (BAASIS©): a validated self-report measure assessing adherence to implementation issues (e.g., drug holidays; 4 items; yes/no) and persistence/discontinuation (1 item; yes/no) [87].	Paper survey (pat.)
	(3) Daily intake (date and time) of immunosuppressive medication will be monitored electronically via the MEMS® Button, [86] which has been indicated previously as the preferred electronic monitoring device [63]. Patients will receive the button shortly before discharge and are instructed on how to use it at home until their immunosuppressants are discontinued. Data will be password-protected and stored on the MEMS® Adherence Software database, which provides an overview of the electronically compiled dosing history per patient [86].	MEMS® Button [86]
Treatment burden	German version of the PETS© self-reporting questionnaire [88]: nine multi-item domain scales, each measuring the burden of one aspect of chronic illness treatment on a 4- or 5-point Likert-type response scales regarding a 4-week recall time frame: medical information (7 items); medications (7 items); medical appointments (3 items); health monitoring (2 items); interpersonal challenges (4 items); health care expenses (5 items); difficulty with health care services (7 items); role/social activity limitations due to self-management (6 items); and physical/mental exhaustion due to self-management (5 items). Raw domain scores are transformed to a standardised 0-to-100 metric, with higher scores indicating greater treatment burden.	Paper survey (pat.)
Health-related Quality of Life	Measured using the EQ-5D-5L© [89], covering five dimensions (mobility, self-care, usual activities, pain/discomfort and anxiety/depression) and including the EQ–Visual Analogue Scale (VAS), on which individuals rate their overall perceived health state (scale of 0 to 100)	Paper survey (pat.)
Quality-Adjusted Life Year (QALY)	Calculate QALY: Generating these requires the HRQL preference weight (obtained from the EQ-5D-5L© value set for Germany EQ-VT v. 2.0) [92] and time in days between HRQL measurements [93]. QALY scores range from 1 (perfect health) to 0 (dead) [94].	Paper survey (pat.)
GvHD	Incidence and grade of chronic and acute GvHD	EHR
Overall survival rate	Overall survival rate from start of study participation	EHR
Conditioning regimen	Treatments used to prepare a patient for stem cell transplantation (e.g., chemotherapy, monoclonal antibody therapy, and radiation to the entire body)	EHR
Donor match/type	Human leukocyte antigen (HLA) tissue type	EHR
Disease	Primary disease	EHR
If death	Date and cause of death	EHR
If relapse	Date of relapse	EHR

*Abbreviations. BAASIS* Basel Assessment of Adherence to immunosuppressive medication scale. *EHR* Electronic Health Record, *EQ-5D-5L*© European Quality of Life 5 Dimensions 5 Level Version, *EQ-VT* EQ valuation protocol, *GvHD* Graft-versus-Host-Disease, *HRQL* health related quality of life, *pat* patient, *PETS* Patient Experiences with Therapy and Self-management, *QALY* quality-adjusted life years

#### Effectiveness outcomes

The primary effectiveness outcome will be the re-hospitalisation rate. Table 3 summarises primary and secondary effectiveness outcomes and their measurement. Specifically for the Swiss setting [63], medication adherence is assessed in addition to a self-report scale (BAASIS©) also using the MEMS® Button [86] electronic monitoring (EM) device. The latter device being integrated in the study based on a qualitative exploration on patient preferences and patient’s evaluation of its usability in daily life [63].

#### Implementation outcomes and evaluation of implementation pathway

After each of the 12 personal consultations, implementation outcomes (i.e., acceptability, appropriateness, feasibility, and fidelity) will be assessed using quantitative methods via surveys of intervention patients and of the CCs (APNs), who provide the SMILe-ICM (Table 4). The implementation pathway will be evaluated via a qualitative approach after the intervention period with input from the entire alloSCT team and all intervention patients.

**Table 4:** Implementation outcomes

Outcome variable(s)	Measure(s) and data collection procedures	Data source and reporter (when applicable)
**Acceptability from patient’s perspective**	Acceptability—reflecting the end users’ satisfaction with the intervention—will be assessed using the 4-item Acceptability of Intervention Measure (AIM). Each item applies a 5-point Likert-type scale ranging from 1 (completely disagree) to 5 (completely agree) with higher scores indicating greater acceptability [90].	Paper survey (pat.)
**Appropriateness from patient’s perspective**	The intervention’s appropriateness, i.e., its perceived suitability to address problems within its target setting, will be assessed via the 4-item Intervention Appropriateness Measure (IAM). Each item applies a 5-point Likert-type scale ranging from 1 (completely disagree) to 5 (completely agree) with higher scores indicating greater appropriateness [90].	Paper survey (pat.)
**Feasibility from patient’s perspective**	The intervention’s perceived suitability for everyday use – will be assessed with the Feasibility of Intervention Measure (FIM). Each item applies a 5-point Likert-type scale ranging from 1 (completely disagree) to 5 (completely agree) with higher scores indicating greater feasibility [90].	Paper survey (pat.)
**Technology acceptability**	The ratio of the number of data entry days to the number of technological problems, will be a measured using data gathered via the SMILeApp.	SMILe monitoring data base (SMILe Care)
**Acceptability from CC’s perspective**	Acceptability—reflecting the end users’ satisfaction with the intervention—will be assessed using the 4-item Acceptability of Intervention Measure (AIM). Each item applies a 5-point Likert-type scale ranging from 1 (completely disagree) to 5 (completely agree) with higher scores indicating greater acceptability [90].	Paper survey (CC)
**Appropriateness from CC’s perspective**	The intervention’s appropriateness, i.e., its perceived suitability to address problems within its target setting, will be assessed via the 4-item Intervention Appropriateness Measure (IAM). Each item applies a 5-point Likert-type scale ranging from 1 (completely disagree) to 5 (completely agree) with higher scores indicating greater appropriateness [90].	Paper survey (CC)
**Feasibility from CC’s perspective**	The intervention’s perceived suitability for everyday use will be assessed with the Feasibility of Intervention Measure (FIM). Each item applies a 5-point Likert-type scale ranging from 1 (completely disagree) to 5 (completely agree) with higher scores indicating greater feasibility [90].	Paper survey (CC)
**Intervention fidelity**	The gold standard measure of intervention delivery (observations/evaluations using prespecified criteria) [95] will be used: Intervention participants’ attendance as planned to face-to-face visits T0 – T8 (fully, partly or not at all) will be noted by the CC. Any deviation from the intervention protocol will be recorded in view of number, length, frequency of contacts, and delivered content.	CRF (RA)
**Implementation pathway**	To explore potential implementation process barriers and facilitators as well as problems experienced in its delivery, we will conduct focus group interviews with the alloSCT team including 10–15 health care professionals (i.e., haematologists, nurses, psycho-oncologists, management), who are involved in the in-patient and follow-up care, plus 10 individual intervention patient interviews.	Individual interviews (pat.)
		Focus group interviews (health care professionals)

*Abbreviations. CC* Care Coordinator, *pat* patient; *RA* Research Assistant

#### Analysis

Statistical analyses will be conducted at the University of Basel’s Institute of Nursing Science using the R software package [96]. Data cleaning will include systematic screening for out-of-range values and data inconsistencies. Multiple imputations will be considered for missing data. Descriptive statistics will be applied as appropriate for all variables.

- ***Aim 1a:*** To determine whether hospitalisation rates (primary outcome) are reduced via the SMILe-ICM’s implementation, we will analyse any differences between IG and UCG rates by applying generalised linear mixed models (GLMM) [97]. The relevant variables (i.e., time, study group, and group x time interaction) will be entered into the models. Our targeted test statistic is the rate ratio by unconditional maximum likelihood estimation (Wald) as described by Rothman et al. [98] and implemented in epitools [99]. We will conduct intention-to-treat and per-protocol analysis. Two-sided significance will be set at 0.05. To analyze the target secondary outcomes over the course of time, descriptive analyses will be carried out and GLMM will be used to determine differences between the IG and UCG. Cost data will be collected and analysed quarterly. Overall survival will be analysed using the Kaplan-Meier method and the log-rank test. To analyse QALY, we will use the EQ-5D-5L**©** value set for Germany. The electronically compiled dosing history will be analysed using by applying GLMM, which account for dependence among observations from a single patient over time. The resulting estimates will be expressed as odds ratios (ORs) [100].
- ***Aim 1b:*** To test a potential wane-out effect of the SMILe-ICM’s intervention effect over 3 months’ post-intervention follow-up, differences between the IG and the UCG will be determined by applying GLMM. This approach allows calculation of the main group and time effects, and of group-by-time interaction effects. Two-sided significance will be set at 0.05.
- ***Aim 2:*** Acceptability of Intervention Measure (AIM), Intervention Appropriateness Measure (IAM), and Feasibility of Intervention Measure (FIM) (implementation outcomes) [90] will be descriptively evaluated. Independent Student’s t-tests will be calculated to determine whether implementation outcome scores will change significantly within the IG over the study period. Concerning the analysis of the implementation pathway, field notes, audiotapes of interviews and mind maps of focus group discussions will serve as qualitative data. Transcript data will be stored and analysed in the ATLAS.ti 8 software package [101]. Individual interviews with patients will be thematically analysed following Braun et al.’s six-phase procedure—an approach using stepwise systematic and iterative processing of data to arrive at a meaningful description and interpretation [102]. During the focus group interviews with clinicians, key themes will be mind-mapped on a flipchart to help the researcher recall previous thoughts and summarise all of the focus groups’ input. Participants will have the opportunity to reflect on the maps and to add or change keywords [103]. After the final focus group session, all mind maps will be combined into a single meta-map using the Microsoft Visio Professional 2019 software [104]. We will then apply Mayring’s approach to qualitative content analysis [105].

## Discussion

Many European countries, including Switzerland, promote innovation approaches to delivering outpatient care. As the demand for outpatient care grows, there is increasing tension on health care systems, due to volume and cost, based on principles of integrated care [29, 106–108]. The use of technology such as eHealth is common within policy agendas [109, 110]. In addition, emerging evidence regarding its capacity to deliver interventions for both acute and chronically ill patients will potentially accelerate innovation in integrated care delivery. eHealth-facilitated interventions can be effective on health- and cost-related outcome measures [9]. In addition to the potential of eHealth-facilitated interventions to reduce re-hospitalisation rates and shorten length of stay [111–113], such interventions can improve patients’ medical and behavioural outcomes in view of symptom severity, quality of life (QoL), medication adherence [7–11] and other behavioural performance measures [3, 114, 115]. While eHealth-facilitated ICMs have been tested in trial settings, their successful implementation in clinical settings remains a challenge [116–118]. Very limited work has focused on bundled implementation strategies to support their implementation and evaluating of implementation outcomes and implementation pathways is largely lacking. Additionally, it is not known which strategies (or bundles) are appropriate for a particular context.

This research shortfall hinders, delays and may even prevent successful translation and scaling-up of interventions to real-life clinical settings. Therefore, the SMILe–ICM—which, as noted, was developed by combining implementation-, computer-, and behavioural-science methods—will now be tested in a hybrid effectiveness-implementation RCT. If successful, in addition to bridging important gaps in view of clinical practice for alloSCT care, this model’s multi-field combination of elements will represent a major methodological innovation.

SMILe’s implementation will be guided by bundled, contextually-adapted implementation strategies. These strategies were determined by our contextual analysis, empirical evidence, theory, and stakeholder involvement. However, context is not static. On the contrary, context can change rapidly (e.g., personnel and/or leadership can change, a pandemic can strike) potentially defeating the best-prepared process. Our planned evaluation of the SMILe–ICM including the bundle of implementation strategies will use mixed methods to shed light on adaptations to strengthen the SMILe-ICM and the bundle of implementation strategies in future scale-up.

Issues with the model’s technology component may also arise. To counter this possibility, software components have been specifically developed for this project using an agile software development approach [61]. Agile development methodology is an iterative approach: working parts of the software are developed in close collaboration with stakeholders, and feedback is collected early. This enables regular and early user tests, each of which enhances the likelihood of acceptance and fidelity. Additionally, alongside the USB’s IT department, the SMILe project’s software development team has devoted considerable time and expertise to embedding the technology component very well within the USB’s IT ecosystem. Moreover, the SMILe software team will be closely involved in the evaluation of the SMILe-ICM, providing technological support where needed.

As important as any eHealth solution’s health benefits may be, ethical aspects are equally important. For example, to safeguard patients’ autonomy and privacy (121–124), data are pseudonymised, with only APNs having access to the patients’ private data. And patients will always have the possibility to actively block data exchanges pertaining to them, i.e., they can select whether their entered data can be viewed in SMILeCare, the transplant team’s monitoring platform (125). In an affiliated project, we will closely track any negative or ambiguous effects (125–127) of SMILe-ICM. I.e., the DARE project (new DAta new REsponsilities) [119] will provide insights into the ethical and social implications of eHealth solutions in general and SMILe in particular. Comprehensive exploration of this dimension is crucial to future digitalization processes and scaling-up either of the eICM or of any care models that involve eHealth components.

The current study includes some risk of contamination bias: while the SMILe–ICM will be tested in an RCT, after the patients are randomised into UCG and IG, they will receive their care from the same alloSCT team. To minimise this risk, the APNs/CCs will only have contact with the IG participants. While this approach will not fully exclude the possibility of contamination, it will reduce it to an acceptable level. Admittedly, a cluster randomised approach would reduce this risk further; yet, given the importance at this stage of the SMILe–ICM’s development of establishing its effectiveness, the RCT design will be adequate.

## Conclusion

The SMILe–ICM has the potential to bring true innovation to the current alloSCT follow-up approach by re-engineering it into an integrated, comprehensive, multidisciplinary eHealth-facilitated model of care supported by a bundle of implementation strategies. In addition to supporting fast and well-informed reactions to acute, sometimes life-threatening symptoms, the SMILe-ICM will support patients’ self-management. Developed by combining implementation-, computer- and behavioural-science methods, and now being tested using a hybrid effectiveness-implementation RCT, the SMILe–ICM fills important gaps both in alloSCT care and in implementation science methodology. If it proves effective, this implementation science study will have generated sufficient evidence to support translation and scaling up of the SMILe–ICM to other high-risk chronically ill populations and settings.

## Data Availability

Not applicable.

## Abbreviations

AlloSCT: Allogeneic stem cell transplantation
APN: Advanced Practice Nurse
CC: Care coordinator
eCCM: eHealth enhanced Chronic Care Model
eICM: eHealth-facilitated, integrated care model
EHR: Electronic health record
ePROs: electronic Patient Reported Outcomes
GLMM: Generalised linear mixed models
GvHD: Graft versus Host Disease
IG: Intervention group
RA: Research assistant
RCT: Randomised controlled trial
SMILe: Development/Adaptation, Implementation and Testing of an Integrated Model of Care for allogeneic SteM Cell TransplantatIon faciLitated by eHealth – An effectiveness, implementation science study
UCG: Usual care group
USB: University Hospital Basel
